# T1 Glottic Cancer: Does Anterior Commissure Involvement Worsen Prognosis?

**DOI:** 10.3390/cancers12061485

**Published:** 2020-06-06

**Authors:** Giuditta Mannelli, Lara Valentina Comini, Roberto Santoro, Alessandra Bettiol, Alfredo Vannacci, Isacco Desideri, Pierluigi Bonomo, Cesare Piazza

**Affiliations:** 1 Head and Neck Oncology and Robotic Surgery, Department of Experimental and Clinical Medicine, University of Florence, 50134 Firenze, Italy; roberto.santoro@unifi.it; 2 University of Florence, 50134 Firenze, Italy; lv.comini@gmail.com; 3 Department of Neurosciences, Psychology, Drug Research and Child Health, Section of Pharmacology and Toxicology, Tuscan Regional Centre of Pharmacovigilance and Phytovigilance, University of Florence, 50134 Florence, Italy; alessandra.bettiol@unifi.it (A.B.); alfredo.vannacci@unifi.it (A.V.); 4 Radiation Oncology, Azienda Ospedaliero-Universitaria Careggi, 50139 Florence, Italy; isacco.desideri@unifi.it (I.D.); bonomop@aou-careggi.toscana.it (P.B.); 5 Department of Otorhinolaryngology, Maxillofacial and Thyroid Surgery, Fondazione IRCCS, National Cancer Institute of Milan, University of Milan, 20122 Milan, Italy; cesare.piazza@unimi.it

**Keywords:** early glottic cancer, anterior commissure involvement, prognosis, independent prognostic factor

## Abstract

Radiotherapy (RT) and transoral laser microsurgery (TLM) represent the main treatment modalities for early glottic carcinoma. Local failure is notoriously more frequent in T1b glottic cancer in comparison to T1a and T2 tumors. In this scenario, the role of anterior commissure (AC) involvement is still controversial. The aim of the present study was therefore to determine its potential prognostic power in worsening patients’ survival and outcomes. We categorized different tumor glottic fold locations with respect to the involvement of one (T1a) or both vocal cords, with or without AC involvement. We analyzed a retrospective cohort of 74 patients affected by Stage I glottic cancer, treated between 2011 and 2018 by TLM or RT at a single academic institution. There were 22 T1a (30%) and 52 T1b (70%) cases. The median follow-up period was 30 months (mean, 32.09 ± 18.738 months; range, 12–79). Three-year overall survival (OS), disease-specific survival (DSS), recurrence-free survival (RFS), and laryngectomy-free survival (LFS) were compared according to tumor location, extension, and cT category. According to both uni- and multivariate analyses, an increased risk for recurrence in T1b with AC involvement and T1a tumors was 7.31 and 9.45 times, respectively (*p*-values of 0.054 and 0.030, respectively). Among the 17 recurrences, T1b with AC involvement experienced 15 tumor relapses (88.2%), thus significantly affecting both the RFS and LFS in comparison to the other two tumor subcategories (T1a, *p* = 0.028 and T1b without AC involvement, *p* = 0.043). The deteriorating prognosis in the presence of AC involvement likely reflects the need to power the hazard consistency and discrimination of the T1b category when associated with such a risk factor, thus deserving an independent T category.

## 1. Introduction

Glottic squamous cell carcinoma (SCC) represents about 75% of laryngeal malignancies [1] and arises from the glottic plane which, according to the staging manual of the American Joint Committee on Cancer (AJCC), includes three different anatomical subsites: the vocal cords, anterior commissure (AC), and posterior commissure (PC) [2].

The five-year disease-specific survival (DSS) of T1 glottic SCC ranges between 94% and 100%, with a five-year local control of 84% [3,4]. The AC is rarely the site of origin of these tumors, but it is involved in up to 20% of early glottic cancers [5,6]. The AC’s complex anatomy constitutes a single subsite of the larynx which, according to its embryonic origin, presents a vertical extension [7] as well as a *locus minoris resistentiae* due to the absence of the inner perichondrium in correspondence to the intermediate lamina of the thyroid cartilage [8,9]. In this setting, the AC has always been the object of anatomic, diagnostic, and therapeutic controversies in laryngeal oncology. Furthermore, the pooled difference in five-year local recurrence rates between T1 glottic SCC without (T1a) and with (T1b) AC involvement has been reported to rise up to 12%, regardless of the type of treatment adopted (either radiotherapy (RT) or transoral laser microsurgery (TLM), thus making the presence of AC involvement an independent negative prognostic factor for Stage I glottic lesions [10,11,12,13,14,15]. Although several studies have shown a significant association between AC involvement and a higher recurrence rate of glottic SCC [8,10,11,12,13,14,15,16,17], the specific value of the AC anatomical partition is not taken into account by the current TNM staging system as a structure whose involvement plays an independent prognostic role [18].

Since the binary variables for AC involvement has led to inconsistent results in the literature [10,19], the purpose of this study was to propose a more detailed stratification for tumors involving the AC, in order to better assess its prognostic role in early glottic carcinomas and consequently evaluate its effect on survival. The final aim was to understand if early glottic SCC with AC involvement does deserve a different staging category.

## 2. Materials and Methods

### 2.1. Study Population

A retrospective study on patients treated for T1a and T1b glottic SCC between June 2011 and June 2018 at the Departments of Otorhinolaryngology—Head and Neck Surgery and Radiotherapy, University of Florence, Italy, was carried out. This retrospective observational study was approved by the local Ethical Committee with protocol number 16208_oss, and all the procedures were conducted in accordance to the Helsinki Declaration of 1975, as revised in 1983. All of the patients’ clinical charts were reviewed and tumors retrospectively restaged according to the 8th Edition of the AJCC staging system [18]. All the participants provided informed consent before undergoing curative treatment, either TLM or RT. The indications for both therapeutic approaches included the presence of a biopsy-proven glottic primary SCC, staged as cT1aN0 or cT1bN0 (Stage I) according to the preoperative fiberoptic evaluation, radiological imaging by CAT (computed assisted tomography) and/or MRI (magnetic resonance imaging) with contrast medium administration, and intraoperative endoscopic assessment performed to get a biopsy of the lesion. All patients and their therapeutic options were discussed and shared among the members of the local multidisciplinary team (MDT).

The exclusion criteria were as follows: pre- and/or intraoperative clinical and radiological evidence of glottic SCC different from Stage I; PC involvement; patients previously treated for other head and neck cancers or already submitted to any kind of head and neck surgical procedures, including RT and/or CRT (chemoradiation); patients who did not consent to be enrolled in the study; and patients with incomplete clinical chart records and missing data.

The patients’ demographics, including their Charlson comorbidity index (CCI) [20], tumor and treatment characteristics, and survival rates, were recorded and reviewed in a single dedicated database.

All patients underwent diagnostic preoperative assessment including videolaryngoscopy coupled with narrow-band imaging (NBI) (Olympus Medical Systems Corporation, Tokyo, Japan), and intraoperative panendoscopy with 0°, 30°, and 70° rigid telescopes, under general anesthesia in the operating room. This allowed us to carefully examine the AC, with the specific aim of confirming or excluding its potential involvement. In particular, special attention was devoted to 70° rigid endoscopy to rule out potential subcommissural tumor extension. In the case of multiple glottic lesions, separate biopsies were performed to confirm if they were malignant in nature or not. When surgical margins after TLM were close or positive (especially in the case of deep margin involvement), further re-excision was generally planned. In selected cases, a wait-and-see policy was adopted after patient counseling if the excision was believed to be radical and close/positive margins interpreted as an artifact of laser diathermal effect or surgical specimen processing. Adjuvant radiotherapy (RT) was administered in the presence of clinical and/or pathological factors that increase the risk of tumor recurrence after primary surgical treatment.

On the other hand, primary RT was chosen upon patient request, major concerns regarding vocal outcomes, in cases with a preoperative Laryngoscore ≥9 [21], or whenever unfavorable laryngeal exposure had been confirmed during the pretreatment general anesthesia for bioptic purposes alone. In these cases, a CT scan (Big Bore, Philips Medical Systems, Cleveland, OH, USA) was acquired at a 3-mm slice width for radiation treatment planning. Personalized thermoplastic head, neck, and shoulder masks were created for all patients. A three-dimensional, conformal technique was employed: radiation was delivered by means of opposing lateral fields with proper wedge filters using 6 MV photons. The clinical target volume (CTV) consisted of the whole larynx. A 5-mm CTV to planning target volume (PTV) was applied in order to compensate for potential systematic and random errors. The prescribed dose for the PTV was 70 Gray (Gy) given in 35 fractions of 2 Gy in 7 weeks.

### 2.2. Novel Classification Proposal

**Figure 1:**
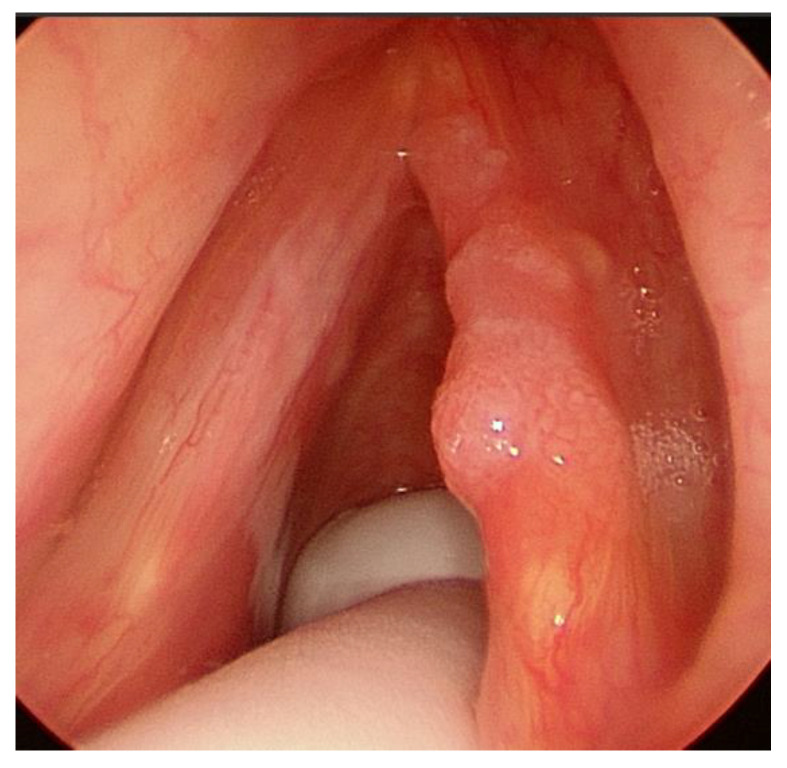
T1a cancer of the right vocal cord without anterior commissure (AC) involvement.

**Figure 2:**
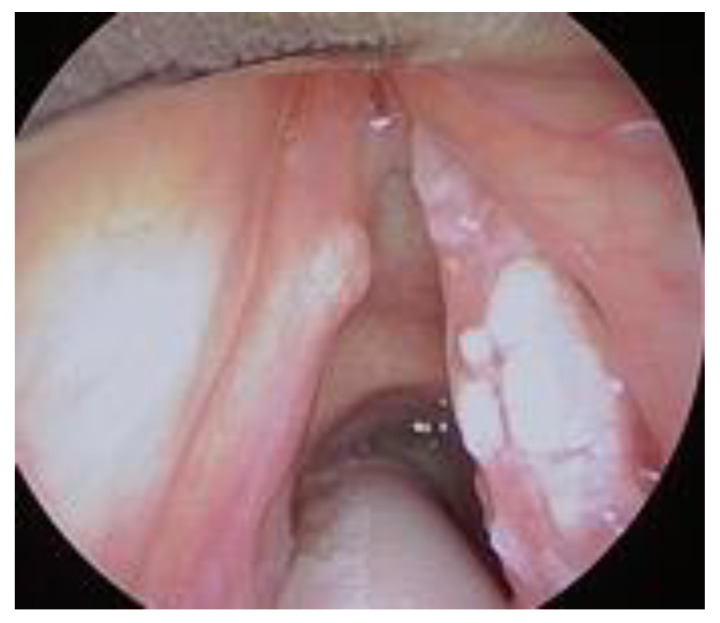
T1b without AC involvement.

**Figure 3:**
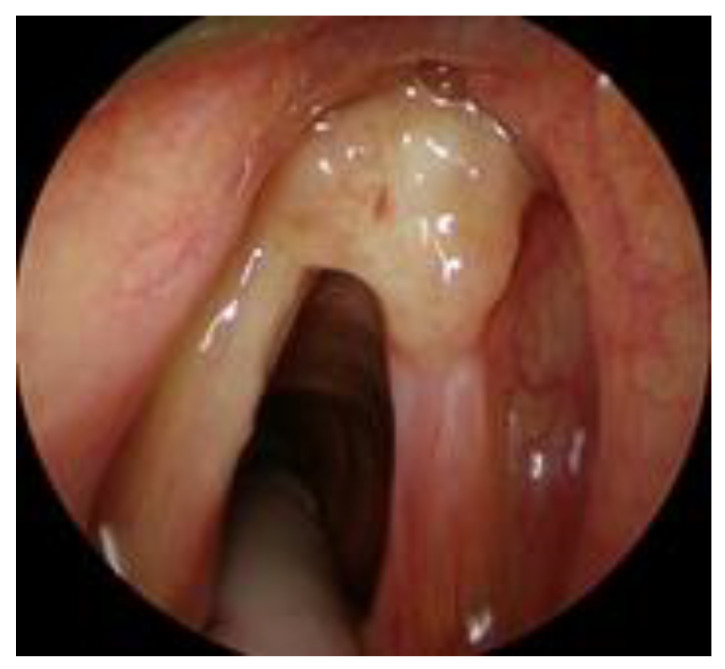
T1b with AC involvement.

To better assess the role of AC involvement in affecting Stage I survival and outcomes, we applied a novel classification system for T1 glottic SCC. AC involvement here refers to all of Rucci’s categories except for the AC0 class [7]. Our proposal is therefore articulated as follows:

1. T1a, tumor limited to one vocal cord, without AC involvement, with normal vocal cord mobility (Figure 1);
2. T1b without AC, tumor involving both vocal cords, without AC involvement, with normal vocal cord mobility (Figure 2);
3. T1b with AC, tumor involving one or both vocal cords, with AC involvement, with normal vocal cord mobility (Figure 3).

### 2.3. Statistical Analysis

The following endpoints were considered: overall survival (OS), defined as the time between the date of surgery and the date of death/last visit; disease-specific survival (DSS), defined as the time between the date of surgery and the date of cancer-related death/last visit; recurrence-free survival (RFS), defined as the time between the date of surgery and the date of recurrence/last visit; and laryngectomy-free survival (LFS), defined as the time between the date of surgery and the date of total laryngectomy/death/last visit.

The influence of the three different abovementioned tumor categories on the prognosis was estimated through the computation of the OS, DSS, RFS, and LFS curves using the Kaplan–Meier method and comparison by the log-rank test for the dichotomic variables. Multivariate analysis was performed using the Cox proportional hazard models and expressed as hazard ratios (HRs) with 95% confidence intervals (CIs).

All the tests were 2-tailed, and *p*-values < 0.05 were considered to be statistically significant. Data were analyzed using Stata 14.0 software (StataCorp LP, College Station, TX, USA).

## 3. Results

Among the 85 patients treated for Stage I glottic SCC by the Head and Neck MDT of our Institution, 74 were enrolled in the present study. Those excluded (*n* = 11) had already received previous head and neck treatments (*n* = 4), presented with PC involvement (*n* = 3), had incomplete pretreatment diagnostic data (*n* = 3), or did not give consent to be enrolled in the present study (*n* = 1). Table 1 summarizes the study population characteristics. A total of 22 T1a (30%), 5 T1b without AC involvement (7%), and 47 T1b with AC involvement (63%) were analyzed. The male to female ratio was 8:1 and the median age at presentation was 68 years (mean, 67.83 ± 11.44; range, 38–90). We did not find any statistical significance in the age categories (<50 years; 50–65 years; >65 years), or in the smoking and alcohol habits among the three tumor categories (T1a, T1b without AC involvement, and T1b with AC involvement). On the other hand, the CCI seemed to be slightly higher in T1b with AC involvement (*p* = 0.049).

Thirty-six patients (48%) underwent TLM, whilst the remaining 38 (52%) received curative RT. The vast majority of T1a patients (90.9%) had surgical treatment, while 66% of T1b with AC involvement underwent RT. This treatment modality represented the preferred therapeutic approach in the presence of AC involvement (*p* < 0.001). The incidence of post-treatment complications, as well as the need for adjuvant RT after primary TLM, showed no statistically significant association with any of the proposed T1 subcategories.

The median follow-up period was 30 months (mean, 32.09 ± 18.73 months; range, 12–79). The three-year OS, DSS, RFS, and LFS were compared according to tumor location, extension, and cT category.

The total number of recurrences was 17 out of 74 patients (22.9%) and salvage treatment was represented by TLM in 7 patients out of 17 (41.2%), salvage total laryngectomy in 7 (41.2%), open partial horizontal laryngectomy in 2 (11.7%), and RT in 1 (5.9%). The recurrence rate was significantly higher among T1b with AC involvement (31.9%) in comparison to T1a (4.5%) and T1b without AC involvement (20%), with a *p*-value of 0.028 (Table 2).

In fact, T1b with AC involvement experienced 15 of 17 total recurrences (88.2%), significantly affecting the RFS and LFS in comparison to the other two (T1a, *p* = 0.028 and T1b without AC involvement, *p* = 0.043).

The OS was 95.5%, 100%, and 95.7% for T1a, T1b without AC involvement, and T1b with AC involvement, respectively. The mortality rate did not show any statistically significant association with either clinical and tumor characteristics or with the type of primary treatment. Specifically, disease-related mortality was found only in the T1b with AC involvement group, although without any statistical significance (*p* = 1.000) (Table 3).

**Figure 4:**
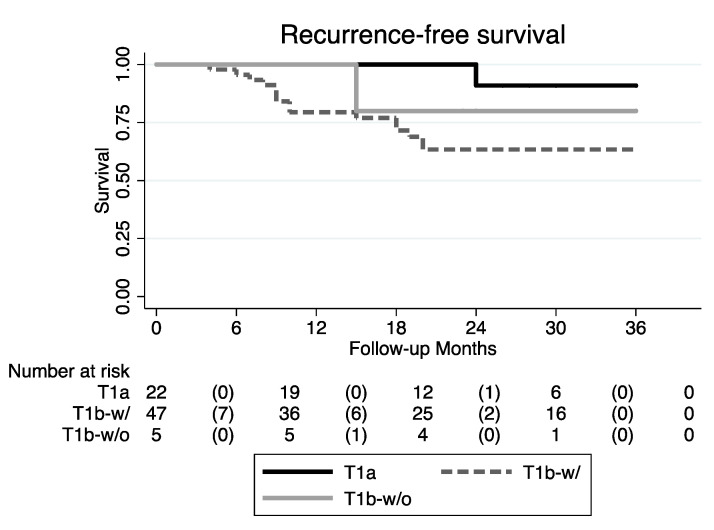
RFS Kaplan–Meier curves (T1b-w/: T1b with AC involvement; T1b-w/o: T1b without AC involvement).

According to both the uni- and multivariate analyses, the increased risk for recurrence in T1b with AC involvement and for T1a tumors was 7.31 and 9.45 times, respectively (*p*-value of 0.054 and 0.030, respectively). Conversely, the risk of recurrence was comparable among patients affected by T1a and T1b without AC involvement (*p* = 0.304) (Figure 4; Table 4).

As reported above, the significant association between T1b with AC involvement and high CCI was also confirmed for the recurrence risk at both uni- and multivariate analyses (*p* = 0.030 and *p* = 0.023, respectively). Again, the type of treatment did not show any statistically significant difference in the recurrence rate among the three tumor categories considered herein in the univariate analysis (*p* = 0.491) (Table 4).

**Figure 5:**
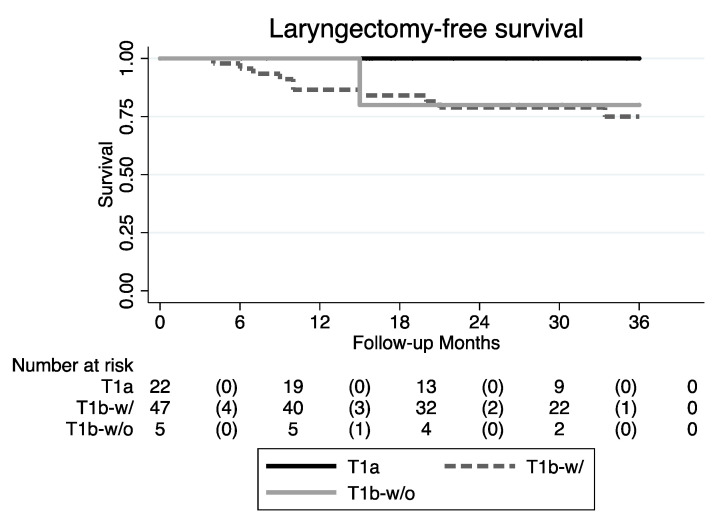
LFS Kaplan–Meier curves (T1b-w/: T1b with AC involvement; T1b-w/o: T1b without AC involvement).

The LFS showed a statistically significant difference (*p* = 0.043) among the three tumor classes (Figure 5 and Table 3).

## 4. Discussion

Although it is widely acknowledged that the presence of AC involvement can have a negative impact on the oncologic outcomes of early glottic SCC, the results so far reported in the literature about its predictive value have been inconsistent, and have generated much debate [19,22,23,24,25]. Even though several reviews have analyzed this issue, none of them definitively solved the existing controversy of the prognostic value of AC involvement [19]. In fact, the unique AC anatomical characteristics make this laryngeal subsite a challenging area for adequate pretreatment endoscopic and imaging workup by either CT or MR (with a high incidence of false negatives adequately assessed only intraoperatively, after surgical resection, or at longer follow-up), difficult area for surgical resection due to laryngeal exposure, and prone to technical radiotherapy issues at the air–tumor interface within the AC due to its variable degree of vascularization, ossification, and three-dimensional conformation/development further complicating its proper management [6,8].

To further elucidate the impact of AC involvement in early glottic SCC in this setting, we started from the assumption that if the extension to the AC represents a worsening prognostic factor, it would definitely deserve a specific T category different from the already existing categories of T1 to which it is usually associated in terms of survival. To accomplish this purpose, we did not linger on T1 spreading patterns or on a proper AC classification system as previously proposed by several authors [7,22]. Instead, we aimed to highlight the potential predictive value of AC involvement by differentiating the existing T1b category, and looking for similarity in prognosis among T1a and T1b without AC involvement (*p* = 0.304) (Table 4), based on our proposed tumor staging and independently for the primary treatment chosen, either surgical (TLM) or non-surgical (RT).

**Figure 6:**
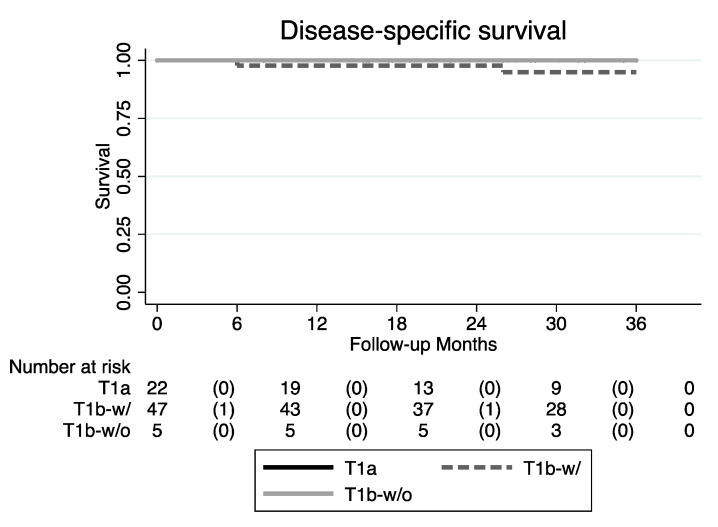
DSS Kaplan–Meier curves (T1b-w/: T1b with AC involvement; T1b-w/o: T1b without AC involvement).

Our study population presented demographics and therapeutic indications comparable to those reported in the literature [10,12]. Even our reported recurrence rates are in accordance with the literature results, which usually range from 17% to 24%, depending on the follow-up schedules and modality [13,14,26]. Interestingly enough, our data confirm that one of the two T1b subcategories (i.e., T1b with AC involvement) represents an independent prognostic factor for survival and resulted in a significant association with a higher recurrence rate in comparison to T1a (*p* = 0.028). By analyzing this prognostic impact on survival, we found a trend towards worse DSS for T1b with AC involvement, though without a statistically significant *p*-value due to the relatively small sample size (Table 3; Figure 6).

The predictive potential of the T1b with AC involvement category was reflected by an even more significant difference in the LFS, which was reported to be 100% in the T1a category, 80% in the T1b without AC involvement, and 76.6% in T1b with AC involvement (*p* = 0.043). These values are also in accordance with the current literature [15,16,17,27,28]. Furthermore, as long as different treatment strategies seem to result in equal clinical outcomes, the matter of post-treatment morbidity and long-term sequelae should be emphasized even more within the decision-making process.

Interestingly, no statistically significant difference were revealed among the three tumor classes, regardless of the respective therapeutic (surgical vs. non-surgical) modality applied, thus reflecting the fact that tumor stage is per se an independent prognostic factor for survival and that choosing the appropriate treatment regimen in the management of AC involvement should be guided by mere functional considerations more than anatomical ones. Notwithstanding, an independent and specific tumor category such as the T1b with AC involvement proposed here would play a role in alerting the treating MDT about the potentially higher risk of suboptimal local control in such a condition.

We do believe that the present classification of T1, which does not take into proper account the AC involvement, might not be fully consistent with the AJCC UICC staging system principles which aim to distinguish the outcomes of tumors by a non-contradictory single prognostic language (https://cancerstaging.org). For these reasons, we suggest that the T staging of glottic SCC should consider AC involvement by a more detailed tumor subsite stratification. Further studies should overcome the limitations of the present analysis, which are mainly due to a small sample size of T1b without AC involvement (also due to their intrinsic rarity), disabling proper and significant statistics. In addition, the application of a prospective study protocol design will give more consistency and allow a more detailed and homogeneous diagnostic imaging acquisition, treatment modalities outcome, and side effects analysis, which was not the main purpose of the current retrospective study.

## 5. Conclusions

In our study, the AC represents the glottic subsite with the greatest risk of local treatment failure and tumor persistence after either TLM and RT [29,30]. Our proposed classification system for glottic T1 with AC involvement could represent a starting point to overcome the actual T1 staging and treatment heterogeneity, where T1b with AC involvement could deserve a separate subcategory due to its evident worse prognosis in comparison to T1a tumors. However, before including such an AC subclassification system into future editions of the AJCC UICC staging system, multicenter, large-sampled cohorts of T1 with different extensions, treated by either TLM or RT, need to be prospectively collected and evaluated in order to confirm our preliminary data.

## Figures and Tables

**Table 1:** Study population characteristics.

	T1a *n* (%)	T1b without AC Involvement *n* (%)	T1b with AC Involvement *n* (%)	*p*-Value (* Statistically Significant)
Number of patients	22 (30)	5 (7)	47 (63)	
Male gender	21 (95.5)	4 (80.0)	42 (89.4)	0.405
Age at diagnosis				
<50 years	0	1 (20.0)	4 (8.5)	0.56
50–65 years	5 (22.7)	3 (60.0)	19 (40.4)	
>65 years	17 (77.3)	1 (20.0)	24 (51.1)	
Charlson comorbidity index (median (IQR))	3 (2–5)	2 (2–2)	3 (2–4)	0.049 *
Smoking habit				
Yes	13 (59.1)	3 (60.0)	31 (66.0)	0.267
No	8 (36.4)	1 (20.0)	8 (17.0)	
Missing data	1 (4.6)	1 (20.0)	8 (17.0)	
Alcohol abuse				
Yes	3 (13.6)	1 (20.0)	8 (17.0)	0.495
No	18 (81.8)	3 (60.0)	31 (66.0)	
Missing data	1 (4.6)	1 (20.0)	8 (17.0)	
**Disease characteristics**				
Tumor location				
Free margin	19 (86.4)	2 (40.0)	29 (61.7)	0.015 *
Superior aspect	3 (13.6)	3 (60.0)	8 (17.0)	
Inferior aspect	0	0	10 (21.3)	
**Treatment**				
Type of treatment				
Transoral laser microsurgery (TLM)	20 (90.9)	0	16 (34.0)	<0.001 *
Radiotherapy (RT)	2 (9.1)	5 (100.0)	31 (66.0)	
Resection margin				
Negative	17 (85.0)	-	12 (75.0)	0.530
Positive superficial, single	1 (5.6)	-	0	
Positive superficial, multiple	0	-	1 (6.3)	
Positive deep, single	0	-	1 (6.3)	
Positive deep, multiple	0	-	1 (6.3)	
Missing data	2 (10.0)		1 (6.3)	
Post-treatment complications				
No	15 (68.2)	2 (40.0)	35 (74.5)	0.249
Yes	7 (31.8)	3 (60.0)	12 (25.5)	
Dysphagia	2 (9.1)	1 (25.0)	6 (12.8)	
Dysphonia	0	1 (25.0)	0	
Dyspnea	0	1 (25.0)	0	
Stenosis	0	0	1 (2.1)	
Granuloma	5 (22.7)	0	1 (2.1)	
**Adjuvant RT**				
No	21 (95.5)	5 (100.0)	33 (70.2)	0.83
Yes	0	0	5 (10.6)	
Missing data	1 (4.6)	0	9 (19.2)	

**Table 2:** Disease recurrence and mortality rates.

	T1a *n* (%)	T1b without AC Involvement *n* (%)	T1b with AC Involvement *n* (%)	*p*-Value (* Statistically Significant)
Number of patients	22 (30)	5 (7)	47 (63)	
**Recurrence**				
No	21 (95.5)	4 (80.0)	32 (68.1)	0.028 *
Yes	1 (4.6)	1 (20.0)	15 (31.9)	
**Salvage treatment for patients with recurrence**				
Redo TLM	1 (4.6)	-	6 (12.8)	1.000
Open partial horizontal laryngectomy	-	-	2 (4.3)	
Total laryngectomy +/- Neck dissection	-	1 (20.0)	6 (12.8)	
RT	-	-	1 (2.1)	
**Disease-related mortality**	0	0	2 (4.3)	1.000
**All-cause mortality**	1 (4.6)	0	2 (4.3)	1.000

**Table 3:** Survival outcomes.

	OS	DSS	RFS	LFS
**T1a**	21/22 (95.5%)	22/22 (100%)	21/22 (95.5%)	22/22 (100%)
**T1b without AC involvement**	5/5 (100.0%)	5/5 (100%)	4/5 (80.0%)	4/5 (80.0%)
**T1b with AC** **involvement**	45/47 (95.7%)	45/47 (95.7%)	32/47 (68.1%)	37/47 (76.6%)
*p*-value	1.000	1.000	0.028 *	0.043 *

OS: overall survival; DSS: disease-specific survival; RFS: recurrence-free survival; LFS: laryngectomy-free survival; * *p*-value statistically significant.

**Table 4:** Univariate and multivariate analyses of risk of recurrence.

	Univariate Analysis		Multivariate Analysis	
	Hazard Ratio (95% CI)	*p*-Value (* Statistically Significant)	Hazard Ratio (95% CI)	*p*-Value (* Statistically Significant)
Tumor extension				
T1a	Ref.			
T1b without AC involvement	3.56 (0.22–57.01)	0.369	4.38 (0.26–73.28)	0.304
T1b with AC involvement	7.31 (0.97–55.37)	0.054	9.45 (1.24–72.30)	0.030 *
Gender				
Male	Ref.			
Female	1.24 (0.28–5.44)	0.772		
Age at diagnosis				
<50 years	Ref.			
50–65 years	2.00 (0.25–16.06)	0.513		
>65 years	1.34 (0.17–10.74)	0.783		
Charlson comorbidity index	0.47 (0.25–0.87)	0.017 *	0.49 (0.26–0.91)	0.023 *
Smoking habit	2.49 (0.56–11.13)	0.233		
Alcohol habit	2.85 (0.95–8.56)	0.062		
Tumor location				
Free margin	Ref.			
Superior aspect	0.77 (0.22–2.69)	0.678		
Inferior aspect	0.36 (0.05–2.77)	0.328		
Type of intervention				
TLM	Ref.			
RT	1.40 (0.53–3.69)	0.491		
Resection margin				
Negative	Ref.			
Positive	1.74 (0.61–4.94)	0.299		
